# The impact of silicon on cell wall composition and enzymatic saccharification of *Brachypodium distachyon*

**DOI:** 10.1186/s13068-018-1166-0

**Published:** 2018-06-20

**Authors:** Sylwia Głazowska, Laetitia Baldwin, Jozef Mravec, Christian Bukh, Thomas Hesselhøj Hansen, Mads Mørk Jensen, Jonatan U. Fangel, William G. T. Willats, Marianne Glasius, Claus Felby, Jan Kofod Schjoerring

**Affiliations:** 1 Department of Plant and Environmental Sciences, University of Copenhagen, Thorvaldsensvej 40, 1871 Frederiksberg C, Denmark; 2 Department of Chemistry and INANO, Aarhus University, Langelandsgade 140, 8000 Aarhus C, Denmark; 3 Department of Geosciences and Natural Resource Management, University of Copenhagen, Rolighedsvej 23, 1958 Frederiksberg, Denmark

**Keywords:** *Brachypodium distachyon*, Silicon, Cell wall composition, CoMPP, Recalcitrance, Hydrothermal pretreatment, Bioenergy

## Abstract

**Background:**

Plants and in particular grasses benefit from a high uptake of silicon (Si) which improves their growth and productivity by alleviating adverse effects of biotic and abiotic stress. However, the silicon present in plant tissues may have a negative impact on the processing and degradation of lignocellulosic biomass. Solutions to reduce the silicon content either by biomass engineering or development of downstream separation methods are therefore targeted. Different cell wall components have been proposed to interact with the silica pool in plant shoots, but the understanding of the underlying processes is still limited.

**Results:**

In the present study, we have characterized silicon deposition and cell wall composition in *Brachypodium distachyon* wild-type and *low-silicon 1* (*Bdlsi1*-*1*) mutant plants. Our analyses included different organs and plant developmental stages. In the mutant defective in silicon uptake, low silicon availability favoured deposition of this element in the amorphous form or bound to cell wall polymers rather than as silicified structures. Several alterations in non-cellulosic polysaccharides and lignin were recorded in the mutant plants, indicating differences in the types of linkages and in the three-dimensional organization of the cell wall network. Enzymatic saccharification assays showed that straw from mutant plants was marginally more degradable following a 190 °C hydrothermal pretreatment, while there were no differences without or after a 120 °C hydrothermal pretreatment.

**Conclusions:**

We conclude that silicon affects the composition of plant cell walls, mostly by altering linkages of non-cellulosic polymers and lignin. The modifications of the cell wall network and the reduced silicon concentration appear to have little or no implications on biomass recalcitrance to enzymatic saccharification.

**Electronic supplementary material:**

The online version of this article (10.1186/s13068-018-1166-0) contains supplementary material, which is available to authorized users.

## Background

The grass family (Poaceae) includes numerous plants species important worldwide as sources of food, feed and bioenergy. One of the distinctive features of grasses is their ability to accumulate silicon (Si) in high quantities, usually over 2% of the shoot dry weight [1]. Multiple studies suggest that Si deposition is correlated with the presence of Si transport proteins (LSI) mediating uptake of orthosilicic acid [Si(OH)_4_] and Si translocation to shoots [2, 3]. Silicon deposited in spines, trichomes and beneath the cuticle in the leaf blades increases the physical resistance of plants towards harmful environmental factors [4, 5]. Based on observations in ex vivo experiments showing that certain cell wall polymers added to silicic acid solution favoured precipitation of Si aggregates, cell wall polysaccharides have also been suggested to interact with Si [6, 7]. The most recent indications of Si involvement in cell wall structures *in planta* come from studies of two Si accumulating species, rice and *Equisetum* [8, 9]. Since plants belonging to both the Poales and Equisetales order contain mixed-linkage glucans (MLG), it was suggested that MLG might serve as a template for Si polymerization in plants [10]. Studies on rice cell wall mutants showed positive correlation between the Si content in biomass and cellulose, hemicelluloses and lignin [11]. In addition, Si supply stimulated deposition of cellulose and hemicelluloses in rice, suggesting further involvement of Si in the formation of cell walls [11]. Hemicelluloses were identified to be ligands binding Si in cell walls of rice cell cultures [9, 12, 13]. In cell walls of fern (*Adiantum raddianum*), Si co-localized with epitopes of homogalacturonans (HG) and 1,5-arabinans [14]. Despite multiple sources of evidence supporting the presence of Si within the cell wall matrix, the nature of interactions with cell wall polymers and the exact role of Si in the process of cell wall assembly remain poorly understood.

Plants benefit from Si uptake and Si fertilization of many crop species is carried out as a standard procedure in agriculture and horticulture [15, 16]. Silicon amendment substantially improves plant growth and vigour upon exposure to stress conditions [17]. On the other hand, the presence of Si in plant material is problematic in industrial utilization of biomass for energy, as it may form insoluble precipitates and cause increased wear of equipment [18]. In addition, cell wall-bound Si may contribute to the recalcitrance of biomass [18]. However, the mechanisms triggering Si deposition within cell walls remain unexplained, and the fact that Si interacts with multiple polymers inside cell wall network makes it very difficult to remove and study [19].

Cell walls are dynamic structures of which the composition and spatial arrangement vary not only among plant species or tissue types, but also changes during plant development and in response to external factors [20]. Secondary cell walls of grasses are formed by cellulose microfibrils embedded in a network of hemicelluloses, hydroxycinnamic acids and lignin with minor contribution of pectins and structural proteins [20]. In addition, mineral elements such as boron (B) and calcium (Ca) play crucial structural roles in the assembly of the cell wall network by cross-linking pectin polymers [21, 22].

Grasses are widely used feedstocks in production of second generation biofuels. To speed up progress in developing bioenergy crops, the model plant *Brachypodium distachyon* has been introduced as a tool for rapid testing of traits and strategies directed towards the use of grasses in bioenergy applications [23]. Features such as short generation time, small genome size, and multiple genetic resources and molecular tools have strengthened the position of *B. distachyon* among other model organisms [23, 24]. The cell wall composition of *B. distachyon* is comparable with several grass species important for agriculture and bioenergy production [25–27]. In addition, the Si uptake system and the levels of Si accumulation in shoot tissues are similar to those in barley, maize and wheat. Together these properties make *B. distachyon* a valuable tool for studies of biomass conversion [28, 29].

In the present work, we have used wild-type and *low*-*silicon 1* mutant (*Bdlsi1*-*1*) plants of *B. distachyon* to unravel the implications of Si on the cell wall composition and the enzymatic digestibility of the biomass. We have studied the organ-specific deposition of Si in the cell walls and its effect upon cell wall polymer profiles at the ripening stage. Compositional changes associated with low Si levels in the mature plants were characterized with the overall intention to link them with the efficiency of enzymatic digestibility. We demonstrate that cell walls of low-Si plants exhibit multiple compositional alterations, most likely affecting the linkage of the individual polymers within the cell wall network. However, as will be shown, these changes have minor or no effects on the saccharification potential of the biomass.

## Methods

### Plant material and growth conditions

Seeds of *Brachypodium distachyon* (accession Bd21-3) wild-type plants and the *low*-*silicon 1* (*Bdlsi1*-*1*) mutant were surface sterilized as previously described [30, 31]. Briefly, the seeds were soaked in MilliQ water for 2 h then washed with 70% (v/v) ethanol for 30 s, 1.3% sodium hypochlorite (v/v) for 4 min and rinsed with sterile MilliQ water. The seeds were vernalized in the dark at 5 °C in MilliQ water for 4 days. Thereafter, the seeds were placed in pots containing soil (Pindstrup Substrate NO. 2; Pindstrup Mosebrug A/S, Ryomgaard Denmark) and transferred to a greenhouse with a temperature around 19 °C and a 16 h photoperiod. The plants were harvested at two developmental stages corresponding to the Biologische Bundesanstalt, Bundessortenamt und CHemische Industrie (BBCH) scale developmental stages of ripening (BBCH 85–89) and senescence (BBCH 92–99) [32]. A total of 9 plants, sampled as 3 pools of 3 plants, were harvested at each stage, the material was frozen in liquid nitrogen and stored at − 80 °C. At the ripening stage, the sampled plants were subdivided into: (i) leaf blades and sheaths; (ii) stems; and (iii) spikelets (heads), which included all reproductive organs, meaning flowers, seeds and bracts (seed covers). At senescence (maturity), entire shoots, including all the above-ground organs, i.e. both the vegetative and generative parts (straw material and all the spikelets containing mature seeds) were harvested and analysed as a whole or as the straw part separately, the latter consisting of leaves with sheaths and stems.

### Cell wall material preparation

Cell wall material (alcohol insoluble residue, AIR) was prepared in biological triplicates as described by [33] with adaptations. Plant material was ground using a mortar and pestle and washed 6 times with 70% (v/v) ethanol for 10 min followed by washing with 100% acetone for 10 min. The pellet was left to dry and AIR was obtained. Starch was removed by enzymatic digestion according to [34]. Briefly, 100 mg AIR was resuspended in 100 mM potassium phosphate, pH 6.8 and digested with α-amylase (0.5 U mg^−1^ AIR, α-amylase from *Bacillus subtilis* Type II A, SIGMA A6814-1MU) for 24 h at room temperature. The pellet was washed 3 times with water and 2 times with 100% acetone and freeze-dried.

### Silicon, boron and calcium concentrations in cell wall material

The analyses were performed on samples harvested at the ripening growth stage and at maturity as described below. AIR material (20–30 mg) was weighed into Teflon microwave digestion tubes followed by the addition of 1.25 mL of 70% (v/v) nitric acid and 500 µL of 15% H_2_O_2_ (v/v). The tubes were then capped and the samples digested in a microwave oven at 242 °C for 25 min (UltraWAVE single reaction chamber microwave digestion system, Milestone Inc., Shelton, CT Multiwave 3000, software version 1.24, Anton Paar GmbH, Graz, Austria). The resulting solution was filtered through a membrane filter having a pore size of 0.22 µm (Q-Max RR 25 mm CA, Frisenette ApS, Denmark) in order to withhold phytoliths. After addition of 0.1 mL of 49% (v/v) hydrofluoric acid and 1 mL of 36% (v/v) hydrochloric acid, the filtrate was incubated overnight at room temperature, followed by dilution with MilliQ water to a final volume of 50 mL. The elemental composition of the samples was measured by inductively coupled plasma optical emission spectrometry (ICP-OES; Agilent 5100, Agilent Technologies, Manchester, UK). Reference material (spinach leaf, NCS ZC73013, China National Analysis Center for Iron and Steel, Beijing, China) was included in the analysis to validate analytical precision and accuracy.

### Labelling of silica surfaces in phytoliths

Silica residues (phytoliths) were recovered from the filters, washed several times with water followed by acetone, and left to air dry. The phytoliths were subsequently suspended in 20 mM PIPES buffer (pH 7) containing 0.125 µM 2-(4-pyridyl)-5-((4-(2-dimethyaminoethylaminocarbamoyl)-methoxy) phenyl) oxazole (PDMPO, LysoSensor Yellow/Blue DND-160, 1 mM in DMSO, Thermo Fisher). Samples were left for 24 h to allow the labelling. PDMPO is used to measure the pH of acidic organelles; however, it has been shown to produce green fluorescence upon interaction with silica surfaces, but not silicic acid [6, 35]. Each sample was examined under a fluorescence microscope (Leica DM5000B).

### Cell wall polysaccharide compositional analysis

Non-cellulosic polysaccharide composition and cellulose content were determined following [36] with modifications. Briefly, de-starched AIR material was hydrolyzed in 2 M trifluoroacetic acid (TFA) for 90 min at 121 °C. TFA releases primarily non-cellulosic polysaccharides; however, also parts of cellulose from regions containing kinks or dislocations will be hydrolyzed. TFA was evaporated and pellet resuspended in 500 µL MilliQ water. The supernatant was filtered with 0.45 µm nylon filters for quantification of monosaccharides in the non-cellulosic fraction, whereas the pellet was collected for analysis of the cellulose content (mostly intact microfibrils). The monosaccharide composition was quantified by high-performance anion-exchange chromatography (HPAEC) on Dionex ICS5000 coupled with pulsed amperometric detection (PAD) using CarboPac PA1 column and guard (2 × 250 mm/2 × 30 mm) (Thermo Fisher Scientific) according to the manufacturer’s instructions (TN280) with modifications. For neutral sugars, samples were eluted with MilliQ water at a column temperature of 17 °C in 75 min at a flow rate of 0.250 mL min^−1^, whereas for uronic acids, a multistep gradient of 30 mM NaOH and 1 M NaOAc in 30 mM NaOH was used for elution at a flow rate of 0.250 mL min^−1^ over 17 min. A solution of 300 mM NaOH was applied post-column to improve the sensitivity of detection during both analyses. The separated monosaccharides were quantified using calibration with monosaccharide standards (l-arabinose, l-rhamnose, d-xylose, d-galactose, d-glucose, d-glucuronic and d-galacturonic acid) (Sigma).

The content of intact cellulose microfibrils in the TFA-resistant pellet was quantified based on the method reported by [37]. The TFA-resistant material was treated with Updegraff reagent (acetic acid: nitric acid: water in the ratio 8:1:2, v/v) at 100 °C for 30 min, which removes residual hemicelluloses and regions of cellulose with dislocations that were not removed during hydrolysis with TFA. The remaining pellet was washed one time with water and 3 times with 100% acetone and air dried overnight. Next, the pellet was hydrolyzed with 72% (v/v) H_2_SO_4_ for 45 min at room temperature to break down intact cellulose fibrils. The released glucose was quantified using the colorimetric anthrone assay as follows: 5 μL of each sample was added to a 96-well polystyrene microtiter plate with 95 μL of water and 200 μL of anthrone reagent (2 mg anthrone mL^−1^ concentrated H_2_SO_4_). The plate was then incubated at 80 °C for 30 min, cooled down and the absorbance measured at 625 nm in a microplate reader (Eon™ High-Performance Microplate Spectrophotometer, BioTek). Cellulose content was determined in triplicate for each sample.

### Comprehensive microarray polymer profiling (CoMPP)

The analysis was performed essentially according to the method reported by [38, 39]. Briefly, 10 mg of AIR was first treated with 50 mM trans-1,2-diaminocyclohexane-*N*,*N*,*N*′,*N*′-tetraacetic acid (CDTA), pH 7.5 for 2 h. The supernatant was collected as a pectin-enriched fraction and the remaining pellet was treated with 4 M sodium hydroxide (NaOH) containing 0.1% (v/v) sodium borohydride for 2 h to obtain a hemicellulose-rich fraction. Each extraction was done in triplicates and pooled to one sample. Each sample was mixed 50/50 with printing buffer (55.2% glycerol, 44% water, 0.8% Triton X-100) and spotted on a nitrocellulose membrane, pore size of 0.45 μm (Whatman, Maidstone, UK) using an Arrayjet Sprint (Arrayjet, Roslin, UK) with two technical replicates and three fivefold dilutions giving 8 spots per sample. The arrays were blocked with phosphate-buffered saline (PBS) containing 5% (w/v) fat-free milk powder, followed by incubation with primary monoclonal antibodies (mAbs) or carbohydrate binding modules (CBMs) for 2 h. The probes used in this study are specific for plant cell wall polymers and listed in Additional file 1: Table S1. After washing with PBS, the arrays were probed with a secondary antibodies conjugated with alkaline phosphatase for 2 h. The arrays were then washed and developed using a 5-bromo-4-chloro-3-indolyl-phosphate (BCIP)/nitro-blue tetrazolium chloride (NBT) substrate and scanned using a flatbed scanner (CanoScan 9000 Mark II, Canon, Søborg, Denmark) at 2400 dpi. The signal strength was quantified using Array-Pro Analyzer 6.3 (Media Cybernetics, Rockville, MD, USA) and converted into a heatmap [38].

### Methylesterification degree of uronic acids

The degree of methylesterification was analysed by saponification of AIR followed by enzymatic oxidation of methanol released by alcohol oxidase, as described by [40, 41] with modifications. Briefly, de-starched AIR (4 mg) was saponified for 1 h at room temperature by suspending in 180 µL of water and 60 µL of 1 M NaOH with shaking. Afterwards, the solution was neutralized with HCl and centrifuged. The supernatant with released methanol (50 µL) and alcohol oxidase (0.05 U in 0.1 M sodium phosphate, pH 7.5) (Sigma) was loaded into a microplate and incubated for 20 min at room temperature with shaking. Next, 100 µL of mix containing 0.02 M 2,4-pentanedione in 2 M ammonium acetate and 0.05 M acetic acid was added to each well and incubated for 10 min at 68 °C. Samples were cooled on ice and absorbance at 412 nm was measured in a microplate reader (Eon™ High-Performance Microplate Spectrophotometer, BioTek). The degree of methylesterification was calculated as the molar ratio of methanol to galacturonic acid (expressed in %). The content of galacturonic acid was quantified previously by HPAEC, as described in the “Cell wall polysaccharide compositional analysis” section.

### Quantification of (1→3;1→4)-β-d-glucan

The amount of (1→3;1→4)-β-d-glucan (mixed-linkage glucan, MLG) in samples was determined by the release of (1→3;1→4)-β-d-glucan oligomers after enzymatic digestion with lichenase, followed by hydrolysis with β-glucosidase. The d-glucose produced was then quantified using a glucose oxidase/peroxidase reagent. The β-glucan (Mixed Linkage) assay kit (Megazyme, Wicklow, Ireland) was used and the quantification of (1→3;1→4)-β-d-glucan was performed as described in the manufacturer’s instructions, except that the method was scaled down to 5 mg of dry AIR. Ground barley and oat flour, provided by the manufacturer, served as reference material. Mixed-linkage glucan content was determined in triplicate for each sample.

### Immunolocalization and phloroglucinol staining

Approx. 1 cm long pieces of 70% ethanol-fixed internodes harvested at the ripening stage of growth were dissected, rehydrated and embedded in 8% agarose in Eppendorf tubes. The blocks were glued on a metallic holder and 100 μm thick sections were generated using the vibratome Leica VT1000 s. The sections were collected in PBS (140 mM NaCl, 2.7 mM KCl, 10 mM Na_2_HPO_4_, and 1.7 mM KH_2_PO_4_, pH 7.5) in small sieves in 24-microwell plate. The sections were blocked by 5% milk in PBS for 30 min, incubated for 1 h with the primary antibody (LM12, PlantProbes) at room temperature in a solution of 5% milk in PBS at the 1:10 dilution, washed two times with PBS and incubated for 1 h in anti-rat secondary antibody conjugated with AlexaFluor488 (Invitrogen) at 1:500 dilution. The sections were then washed two times with PBS stained with Calcofluor (0.1 mg mL^−1^ in PBS), washed and mounted on the glass slide in CITIFLUOR (Agar Scientific) mounting media. The sections were scanned using a Leica SP5 laser scanning confocal microscope (LSCM) equipped with Ar and HeNe lasers. All sections were scanned with the same settings.

The saturated solution of phluoroglucinol (Sigma) in 20% HCl was used to stain lignin (Wiesner stain). The sections were placed in the drop of solution, incubated app. 5 min and directly observed using a Olympus BX41 light microscope.

### Measurement of acetyl bromide lignin

The concentration of acetyl bromide lignin was determined according to the method described by [42]. The AIR samples (~ 20 mg) were incubated for 4 h at 50 °C in 2.5 mL 25% acetyl bromide (v/v) (Sigma) in glacial acetic acid. After complete digestion, samples were cooled down and cleared by centrifugation. Then, 30 μL of supernatant, 40 μL 1.5 M NaOH, 30 μL 0.5 M hydroxylamine and 150 μL glacial acetic acid were loaded into a quartz microplate. The lignin concentration was calculated from the UV absorbance at 280 nm using a molar extinction coefficient of 18.126 g^−1^ L cm^−1^ [43] and a 0.6345 cm path length. The lignin analysis was performed in triplicate for each sample.

### Measurement of monolignol ratios and hydroxycinnamates by analytical pyrolysis

The straw or entire shoots of mature plants were pyrolyzed in duplicates in random order. The samples were prepared by transferring about 100–200 µg of AIR to a solvent- and flame-cleaned pyrolysis tube. Pyrolysis was performed at 500 °C (calibrated as sample received temperature). The pyrolysis temperature was held for 20 s by the automated pyrolysis unit (PYRO, GERSTEL) under a He flow of 51 mL min^−1^. The sample and pyrolysis unit were heated from 40 to 300 °C (720 °C min^−1^, held 0.1 min) prior to pyrolysis and maintained at 300 °C for 1 min after pyrolysis by a thermal desorption unit (TDU, GERSTEL). The transfer line was held at 320 °C and pyrolysates were carried onto the column with a 50:1 split in the programmed temperature vaporizer inlet (CIS4, GERSTEL) held at 300 °C. The pyrolysates were separated and detected by an Agilent GC–MS (7890B GC, 5977A MSD) equipped with an Agilent VF-5 ms (60 m, 0.25 mm, 0.25 µm) column with 5 m integrated guard column. The oven program was 40 °C (5 min), to 100 °C (10 °C min^−1^), then to 280 °C (4 °C min^−1^), and finally to 320 °C (10 °C min^−1^, 10 min), giving a total run time of 70 min. The MS was scanning the 35–500 *m/z* range. Only total ion chromatogram (TIC) peak areas of compounds with no or insignificant co-elution were used in calculations. The TIC peak areas were integrated using Agilent Masshunter Quantitative Analysis (ver. B.07.00) and co-elution was evaluated by deconvolution of the TIC in Agilent Masshunter Unknowns Analysis. All compounds used for calculating monolignol and hydroxycinnamate ratios were identified by authentic standards or published mass spectra [44]. The compounds were grouped according to methoxylation into H, G or S. Monolignol ratios were calculated as the peak area of the specific monolignol in proportion to the total peak area of the three monolignols. Analogously, the hydroxycinnamate ratio was calculated.

### Pretreatments and enzymatic saccharification

Pretreatments and enzymatic saccharification were performed on mature straw and entire shoot samples, following the method described by [39]. Dry material was ground and distributed using an automated sample preparation robotic system (Labman Automation Ltd. at Stokesley, North Yorkshire, UK) followed by microscale pressurized heat treatment, mimicking a hydrothermal pretreatment (in-house build system). Briefly, 27 mg of the dried samples was dispensed into a 96-well aluminium plate and 50 mM sodium citrate buffer (pH 4.8) was added to each sample. The plates were sealed with Teflon tape with a little hole above each well, placed on a heating block and further sealed with a thin aluminium plate and a Teflon plate to ensure a gas tight enclosure. The plates were heated to 120 or 190 °C at a stable pressure and incubated for 15 min and 10 min, respectively. Thereafter, the system was cooled down to room temperature prior to enzyme addition. The pretreated material was mixed with the cellulase preparation Cellic Ctec2 (10 FPU g^−1^ biomass; Novozymes, Bagsværd, Denmark), placed in a plate shaker at 50 °C, 450–600 rpm, and hydrolyzed for 50 h. The hydrolyzed samples were filtered through 0.45 μm filter plates (Pall Corporation, Ann Arbor, MI) and 100 μL of each filtrate was mixed with 100 μL of 5 mM H_2_SO_4_ for sugar determination and quantification using high-performance liquid chromatography (HPLC). Separation and determination of the glucose and xylose in the filtrates were carried out by an Ultimate 3000 HPLC (Dionex, Germering, Germany) equipped with a refractive index detector (Shodex, Tokyo, Japan). The separation was performed in a Phenomenex Rezex ROA column (Phenomenex Inc., Torrance, CA, USA) at 80 °C with 5 mM H_2_SO_4_ as eluent at a flow rate of 0.6 mL min^−1^.

## Results

### Concentrations of non-phytolith silicon and cell wall boron are altered in the *Bdlsi1*-*1* plants

The *low*-*silicon 1* mutant (*Bdlsi1*-*1*) plants used in this study carry a mutation in the Si influx transporter (BdLSI1), localized in the roots. The mutation severely hinders Si uptake and resulted in 93% reduction of the shoot Si content [29]. No phenotypic consequences were observed as the morphology and development of the *Bdlsi1*-*1* plants were similar to the wild-type plants [29]. However, the average weight of the mature de-hulled seeds was reduced in the mutant plants despite the fact that the total yield of spikelets was comparable to that of the wild type [29].

**Fig. 1:**
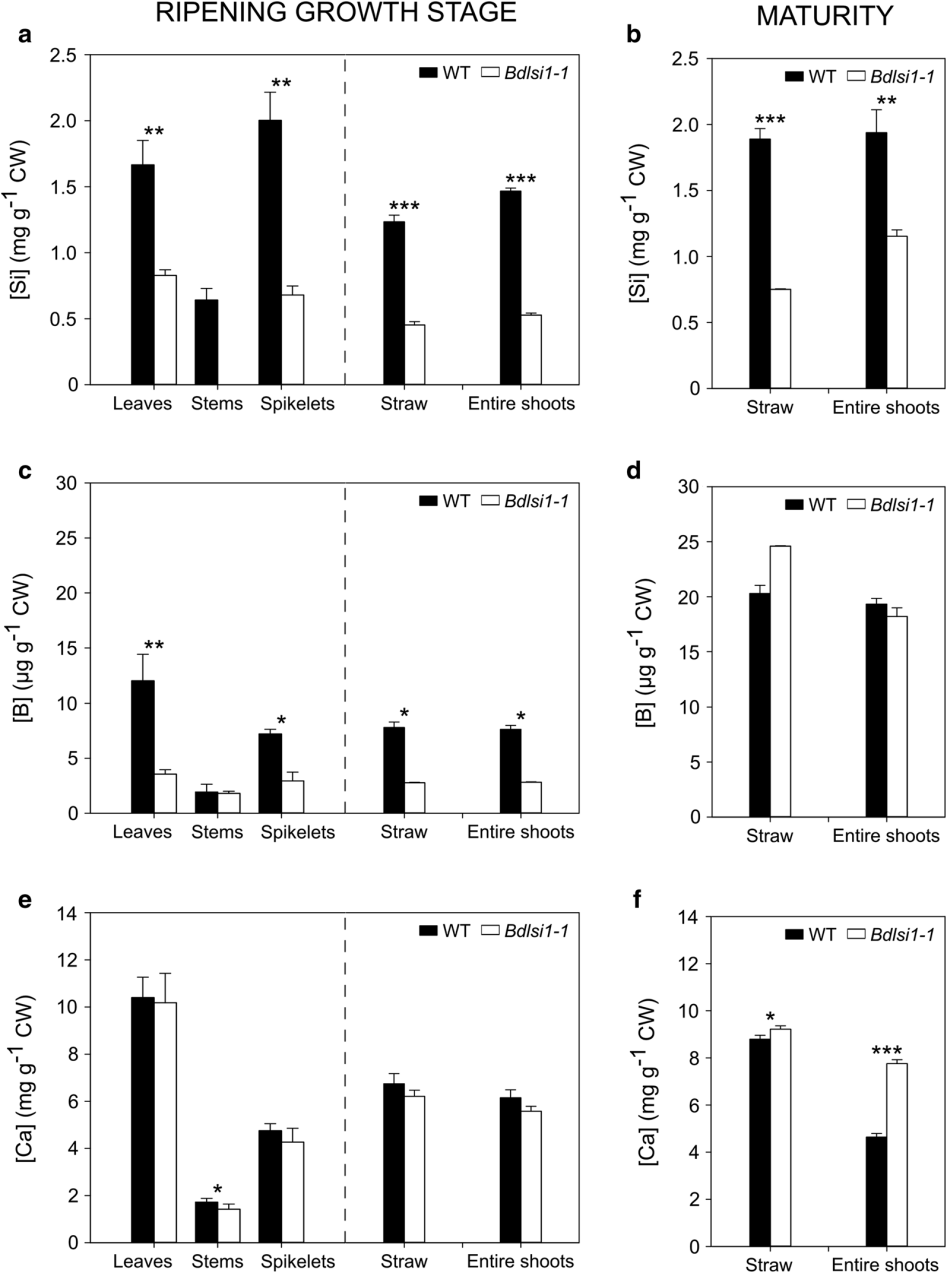
Concentrations of structural mineral elements in cell wall material of wild type (WT) and *Bdlsi1*-*1* mutant plants. Concentrations of non-phytolith silicon, Si (**a**, **b**); boron, B (**c**, **d**) and calcium, Ca (**e**, **f**) in cell walls isolated from different organs of wild-type and *Bdlsi1*-*1* plants growing in soil at 19 °C and 16 h photoperiod in a greenhouse. Material was harvested at two growth stages: (i) ripening (**a**, **c**, **e**) and (ii) maturity (senescence) (**b**, **d**, **f**). At the ripening stage, plants were subdivided into leaves with sheaths, stems and spikelets (flowers, seeds and bracts); data obtained for individual organs were used to calculate element concentrations in straw and entire shoots at the ripening stage (**a**, **c**, **e**). At maturity, either entire shoots (all vegetative and generative above-ground organs) or straw (leaves, leaf sheaths and stems) were harvested. Concentrations were measured by ICP-OES after microwave digestion with acids. Values are mean ± SD of three replicates. Asterisks indicate significant differences between the wild type and the mutant (*p ≤ 0.05; **p ≤ 0.01; ***p ≤ 0.001) as assessed by one-way ANOVA followed by Tukey’s HDS post hoc test

First, we harvested wild-type and mutant plants at the ripening growth stage and at maturity to quantify non-phytolith Si. At the ripening stage, the sampled plants were subdivided into (i) leaf blades and sheaths, (ii) stems, and (iii) spikelets (flowers, bracts and seeds), whereas at maturity either entire shoots (all above-ground organs) or straw only (leaves, leaf sheaths and stems) were collected. Microwave-assisted acid digestion of the wild-type and *Bdlsi1*-*1* plant material resulted in insoluble phytoliths that were isolated by filtration and further labelled with a silica-specific fluorescent dye (Additional file 2: Figure S1). The phytoliths varied in degree of silicification as indicated by the intensity of fluorescence (Additional file 2: Figure S1). The wild-type material contained substantially more phytoliths in all the analysed organs than did the mutant (Additional file 2: Figure S1). The silicon present in the filtrate after acid digestion represented the pool of amorphous Si and Si chemically bound with cell wall polysaccharides, together designated as non-phytolith Si. This pool accounted in all cases for less than 0.2% of the weight of cell walls (Fig. 1a, b). At the ripening stage, the Si concentrations in the leaves and spikelets of the *Bdlsi1*-*1* plants were two and threefold lower, respectively, than in the wild type (Fig. 1a). Despite the overall reduction of non-phytolith Si in the *Bdlsi1*-*1* plants, the distribution pattern between the organs was comparable (Table 1). Both the wild-type and the mutant plants deposited approximately 40% of the shoot Si content in the spikelets and 50–60% in the leaves (Table 1). Significant differences between the wild type and the mutant became evident when comparing the proportion of Si not allocated to phytoliths (Table 1). In the wild-type plants, more than 90% of Si in the entire shoot was associated with phytoliths, while the mutant plants only allocated about 50% of their Si to phytoliths (Table 1). The organ having the largest proportion of Si associated with the cell walls, i.e. non-phytolith Si, in the wild-type plants was the stem. Here, 20% of the Si content was associated with the cell walls against only 11 and 6% in the spikelets and leaves, respectively (Table 1). The mutant plants deviated extensively from this pattern by containing more than 50% of the Si in leaves and spikelets as amorphous or cell wall-bound, while none of the Si in the stems was present in the non-phytolith form (Table 1).

**Table 1:** Silicon distribution among organs and their cell walls in wild-type and mutant plant harvested at the ripening stage

Organ	Plant	Total Si (mg plant^−1^)	Si (% of total Si plant^−1^)	Total Si in cell walls (mg plant^−1^)	Si (% of total Si in CW plant^−1^)	Ratio of Si in CW to total Si (%)
Leaves	WT	40.5	63	2.7	48	6
	*Bdlsi1*-*1*	2.7	51	1.5	58	56
Stems	WT	3.6	6	0.7	13	20
	*Bdlsi1*-*1*	0.7	13	< 0.01^a^	< 1	
Spikelets	WT	20.0	31	2.1	39	11
	*Bdlsi1*-*1*	1.9	36	1.1	42	57
Entire shoot	WT	64.1	100	5.5	100	9
	*Bdlsi1*-*1*	5.3	100	2.6	100	49

Data show the average Si content in different organs of wild-type and mutant plants and the proportion of this Si present in non-phytolith form in cell wall material. The standard deviation of the mean values (*n* = 3) used in the calculations did not exceed 30%

^a^Samples in which Si was below the detection limit (0.01 mg g^−1^ CW)

The concentration of Si associated with the cell walls (i.e. non-phytolith Si) in the straw and entire shoots of wild-type and mutant plants increased between the ripening growth stage and maturity (Fig. 1a, b). The increase in the straw was comparable between the wild type and the mutant (53 and 65%, respectively) (Fig. 1a, b). The concentration of Si associated with cell walls in the entire shoots of the wild type was 32% higher at maturity than at the ripening growth stage, while that in the mutant increased more than twofold between the ripening stage and maturity.

Apart from Si, boron (B) and calcium (Ca) were also quantified as these elements play important structural roles within the cell wall by interacting with pectins. Boron links two molecules of rhamnogalacturonans II (RG-II), whereas Ca forms bridges between non-methylesterified regions of homogalacturonans (HG) [21, 22]. Differences in B concentration between the wild type and the mutant were observed only at the ripening stage, where the concentrations in leaves and spikelets of the *Bdlsi1*-*1* plants were 70 and 60% lower, respectively (Fig. 1c). Calcium concentrations in ripening plants were comparable between the wild type and the mutant in all tested samples, except for the stems, where the mutant had 17% lower Ca concentration than the wild type (Fig. 1e). At maturity, the mutant had 5 and 67% higher Ca concentration in the straw and entire shoots, respectively, than the wild type (Fig. 1f).

### The *Bdlsi1*-*1* mutant exhibits a range of alterations in the composition of non-cellulosic polysaccharides at both the ripening stage of growth and at maturity

**Fig. 2:**
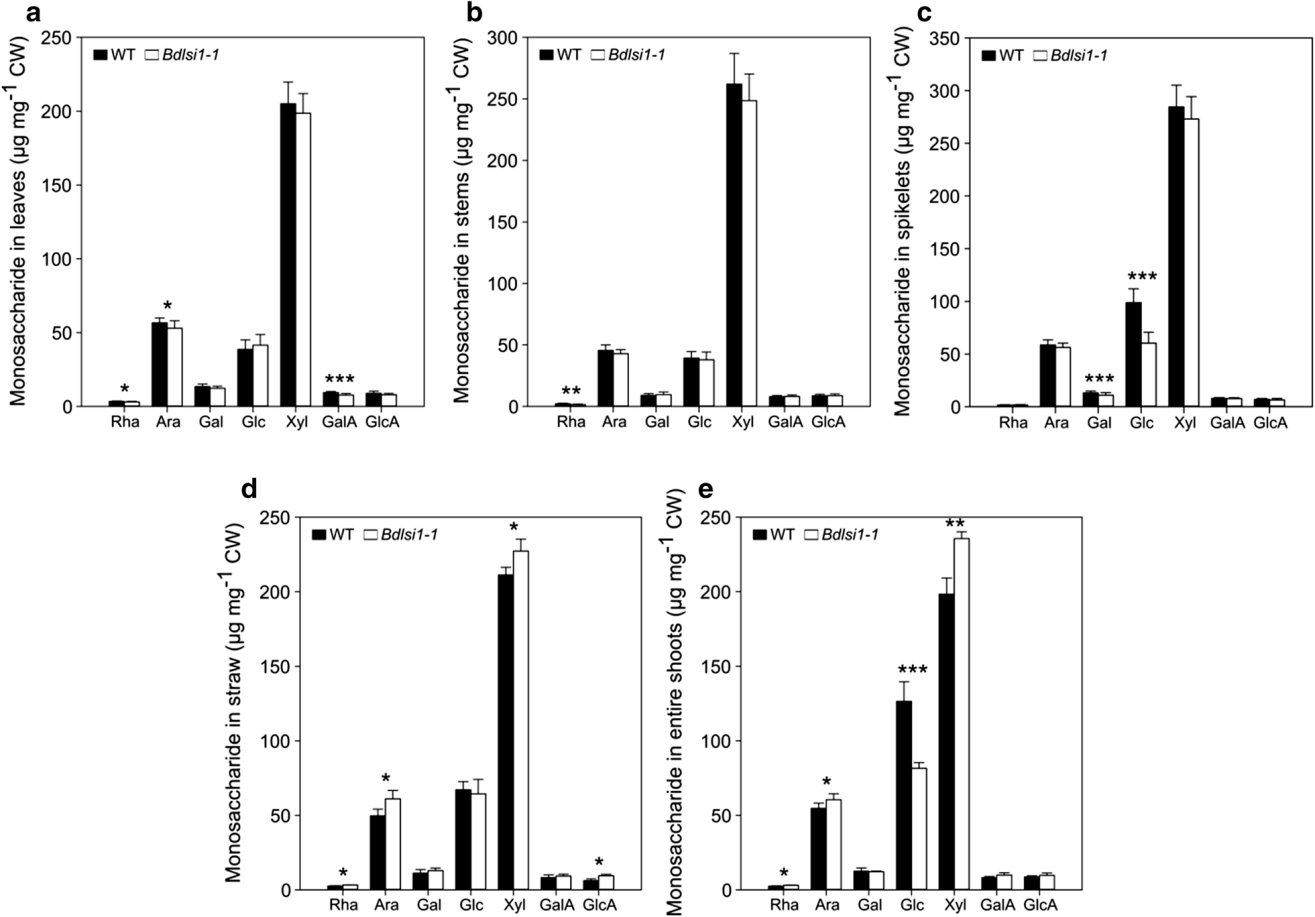
Monosaccharide composition of non-cellulosic polysaccharides in cell walls of wild type (WT) and *Bdlsi1*-*1* plants. Cell walls (CW) were isolated from leaves, stems and spikelets of *B. distachyon* wild-type and *Bdlsi1*-*1* plants harvested at the ripening stage of growth (**a**–**c**) and from straw (**d**) and entire shoots (**e**) at maturity. Samples were obtained by hydrolyzing CW with 2 M TFA, followed by separation and quantification by HPAEC. Data represent mean values (± SD, *n* = 5.) asterisks indicate significant differences (*p ≤ 0.05; **p ≤ 0.01; ***p ≤ 0.001) between the wild type and the mutant as assessed by one-way ANOVA followed by Tukey’s HDS post hoc test. *Rha* l-rhamnose, *Ara* l-arabinose, *Gal* d-galactose, *Glu* d-glucose, *Xyl* d-xylose, *GalA* d-galacturonic acid, *GlcA* d-glucuronic acid

Hemicelluloses and pectins constitute a large part of the cell wall structure and form together a fraction of non-cellulosic polysaccharides [45, 46]. Here, we characterized the composition of monosaccharides associated with pectins (rhamnose, galacturonic acid and galactose) and hemicelluloses (arabinose, glucose, xylose and glucuronic acid) that were released from the cell walls following hydrolysis with trifluoroacetic acid (TFA). The analysis revealed compositional variations among the organs, growth stages as well as alterations between the wild type and the mutant (Fig. 2).

The concentrations of arabinose and galacturonic acid in cell walls of the leaves were significantly lower in the *Bdlsi1*-*1* mutant plants than in the wild type (Fig. 2a). The same was the case for galactose and glucose in the spikelets (Fig. 2c). Rhamnose was the least abundant monosaccharide detected in the cell walls of *B. distachyon* (below 1% of CW) but the concentrations measured in the stems and spikelets at the ripening stage were nevertheless significantly lower in the *Bdlsi1*-*1* plants than in the wild type (Fig. 2b, c). At maturity, rhamnose could still be detected, but the concentrations measured in the straw and entire shoots of the *Bdlsi1*-*1* plants were significantly higher than in the wild type, as was also the case for arabinose and xylose (Fig. 2d, e). Expressed as arabinose to xylose ratio, the mutant had a slightly, but significantly, higher ratio of 0.27 in the straw against 0.22 in the wild type, while in the entire shoots, the values were comparable (0.25). The concentration of TFA-extractable glucose was substantially reduced in the entire shoots of the mutant plants at maturity (Fig. 2e), but not in the straw (Fig. 2d). This corresponded with a decreased concentration of glucose in mutant spikelets at the ripening stage, while the concentrations in leaves and stems were similar to the wild type (Fig. 2a–c).

### The *Bdlsi1*-*1* mutant displays rearrangements in the degree and pattern of homogalacturonan methylesterification

**Fig. 3:**
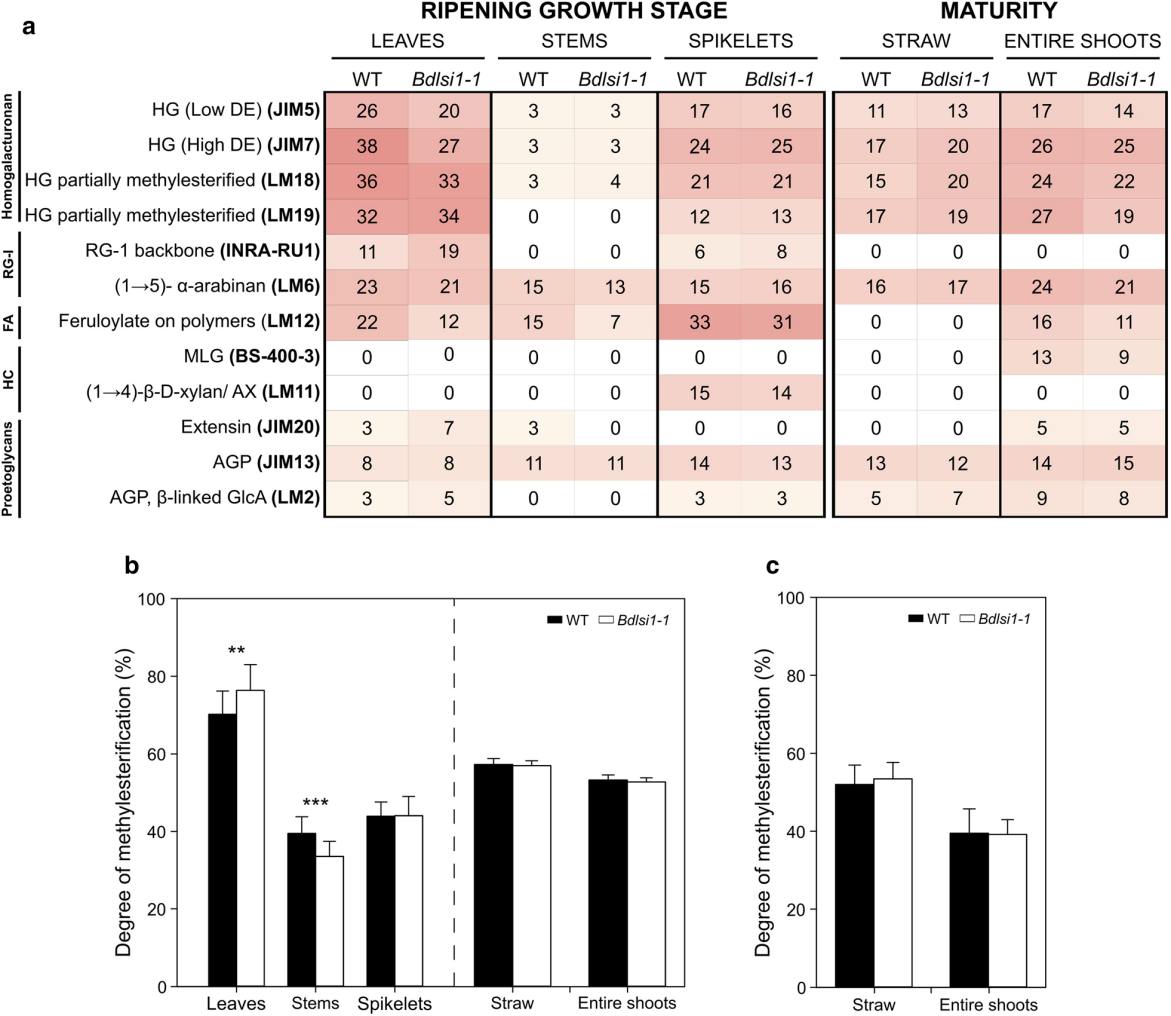
Characterization of the pectin-rich fractions in wild-type (WT) and *Bdlsi1*-*1* plants. Plants were harvested at the ripening stage of growth and at maturity and subdivided as previously described. **a** Comprehensive microarray polymer profiling (CoMPP) showing the relative abundance of polysaccharide epitopes in the pectin-enriched fraction extracted using 1,2-diaminocyclohexanetetraacetic acid (CDTA). The average signal intensities (*n* ≥ 2) are presented as a heatmap in which the white colour corresponds to the lowest intensity and red to the highest. The antibody names and their corresponding epitopes are indicated on the *Y*-axis. Antibodies that did not show signal for all the samples are not included in the heatmap, but listed in Additional file 1: Table S1. The degree of methylesterification of GalA (%) was quantified in the wild type and the *Bdlsi1*-*1* mutant at the ripening stage of growth (**b**) and at maturity (**c**). Data represent the mean ± SD, *n* ≥ 6. All statistical comparisons were made using one-way ANOVA followed by Tukey’s HDS post hoc test, asterisks indicate statistical differences between the wild type and the mutant (**p ≤ 0.01; ***p ≤ 0.001). *HG* homogalacturonan, *RG-I* rhamnogalacturonan-I, *MLG* mixed-linkage glucan, *AX* arabinoxylan, *AGP* arabinogalactan protein, *GlcA* glucuronic acid, *FA* ferulic acid, *HC* hemicelluloses

**Fig. 4:**
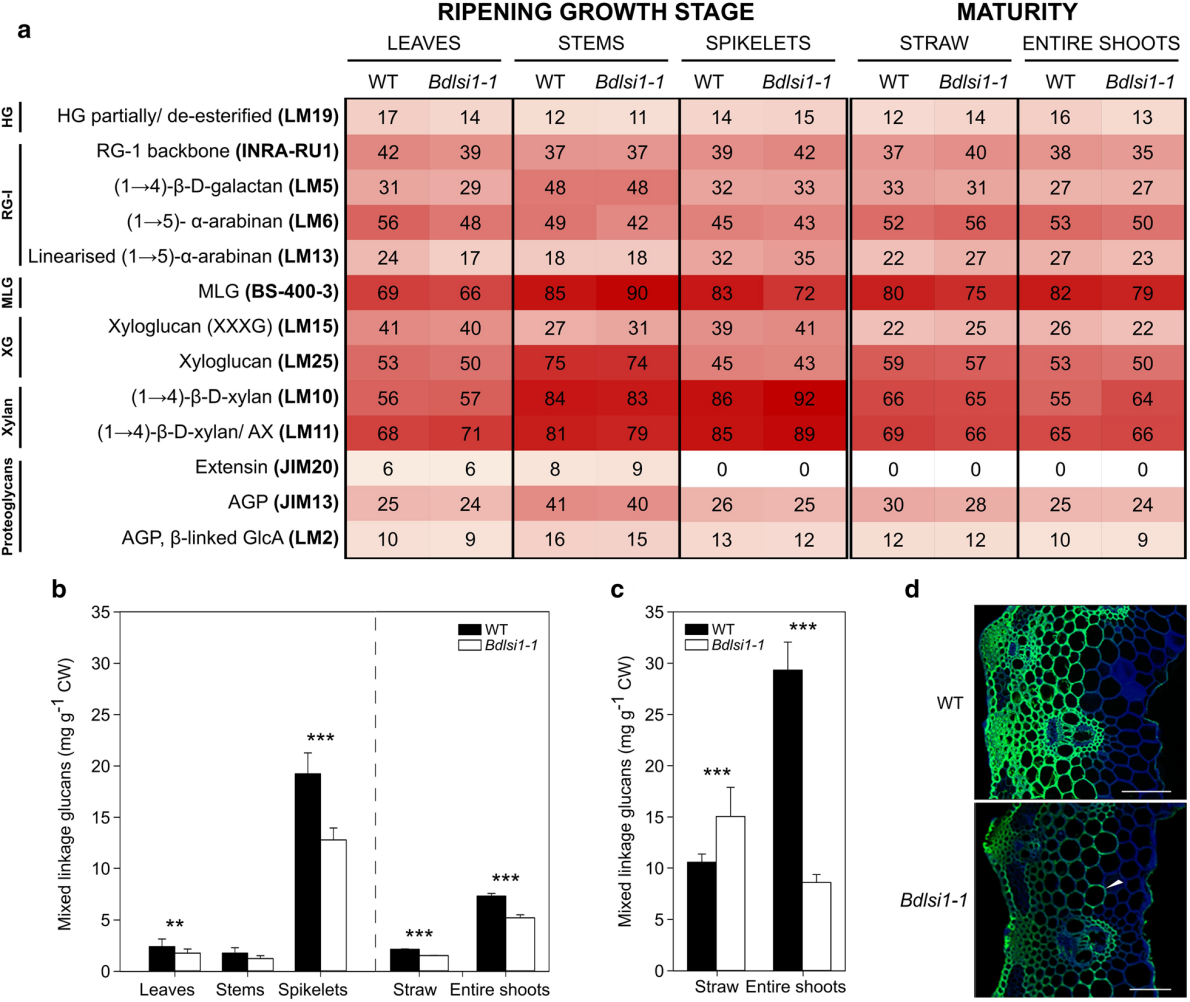
Characterization of polysaccharides in hemicellulose in wild-type (WT) and *Bdlsi1*-*1* plants. **a** Comprehensive microarray polymer profiling (CoMPP) showing the relative abundance of polysaccharides in a hemicellulose-enriched fraction extracted with sodium hydroxide (NaOH) from the wild-type and the *Bdlsi1*-*1* plants harvested at the ripening stage and at maturity. Results are presented as a heatmap of the average antibody signal intensity, where the white colour corresponds to the lowest intensity and red to the highest. The antibodies that did not show signal for all the samples tested are not included in the heatmap (Additional file 1: Table S1). Concentrations of mixed-linkage glucans (MLG) were determined in the wild-type and the *Bdlsi1*-*1* plants at the ripening growth stage (**b**) and at maturity (**c**) using a lichenase-based assay. Data represent the mean (± SD, *n* ≥ 4) and asterisks indicate significant differences (**p ≤ 0.01; ***p ≤ 0.001) between the wild type and the mutant as assessed by one-way ANOVA followed by the Tukey HDS post hoc test. **d** Immunostaining of the feruloylated polysaccharides with LM12 mAb and secondary anti-rat antibody (conjugated to Alexa Fluor 488; green channel) was performed on sections of internodes collected at the ripening stage. Images are overlaid with signal of Calcofluor counterstain (blue) outlining the cell walls. Arrowhead indicates stronger LM12 signal in parenchymatic cells of *Bdlsi1*-*1* stems. Scale bars: 50 μm. *HG* homogalacturonan, *RG-I* rhamnogalacturonan-I, *MLG* mixed-linkage glucan, *XG* xyloglucan, *AX* arabinoxylan, *AGP* arabinogalactan protein, *GlcA* glucuronic acid, *FA* ferulic acid, *HC* hemicellulose

To identify the potential sources of alterations in the individual monomers building the non-cellulosic polysaccharides (Fig. 2), we conducted semi-quantitative comprehensive microarray polymer profiling (CoMPP). This method gives information about extractable cell wall glycans that are released in two consecutive extractions. The first fraction obtained following extraction with cyclohexane diamine tetraacetic acid (CDTA; Fig. 3a) contains predominantly soluble cell wall polymers, especially pectins and proteoglycans, while the second fraction, obtained by extraction with sodium hydroxide (NaOH; Fig. 4a), yields less soluble cell wall components, namely hemicelluloses.

In grasses, pectins account for a relatively low proportion of the cell walls, but their main constituents, rhamnose and galacturonic acid, were nevertheless detected in all of the analysed *B. distachyon* organs (Fig. 2). Several pectin epitopes, mainly structures of homogalacturonans (HG) and rhamnogalacturonan-I (RG-I), were also found in material harvested at both growth stages (Figs. 3a and 4a). Homogalacturonan (HG) is a linear homopolymer of α-(1–4)-linked d-galacturonic acid (d-GalA), which can be further methylesterified at the carbon C6 [47]. In our study, HG was detected with a range of monoclonal antibodies (mAbs) that recognize subtle differences in methylesterification patterns. At the ripening stage, leaves had the highest relative abundance of HG among the organs, and the intensity of several mAbs recognizing HG was lower in the *Bdlsi1*-*1* leaves compared to the wild type (Fig. 3a). The alterations in the relative abundance of the non- and methylesterified HG epitopes detected with CoMPP might reflect changes in the degree and/or pattern of methylesterification as well as in the nature of interactions within the cell wall network. To address this, we measured the degree of methylesterification (DM) of HG using an enzymatic assay. We found that the wild-type and the *Bdlsi1*-*1* plants had comparable DM in all tested samples, apart from the leaves and stems at the ripening stage, where the *Bdlsi1*-*1* had 8% higher and 25% lower DM than the wild type, respectively (Fig. 3b, c). The DM in all the straw samples was comparable between the wild type and mutant and did not change from ripening to maturity (Fig. 3b, c). However, entire shoots of both the *Bdlsi1*-*1* and wild type had about 30% lower DM values at maturity than at the ripening stage of growth (Fig. 3b, c).

We identified the presence of RG-I in *B. distachyon* samples by means of the mAbs directed against the RG-I backbone (INRA-RU1), 1,5-arabinan (LM6 and LM13) and 1,4-galactan side chains (LM5). The epitopes were detected in all the samples, mostly in the fraction extracted with NaOH (Figs. 3a and 4a). In leaves, the abundance of the epitope of the RG-I backbone was much higher in the *Bdlsi1*-*1* samples, while mAbs recognizing both branched and linear arabinose chains showed lower signal relative to the wild type (Figs. 3a and 4a). Several epitopes of AGPs and extensins were detected in both the CDTA and NaOH extractable fractions, suggesting varying nature and strength of their bonds with the cell wall components. However, there were no differences between the *Bdlsi1*-*1* and the wild type plants (Figs. 3a and 4a).

### The concentration of (1→3;1→4)-β-d-glucans is strongly reduced in the spikelets of the *Bdlsi1*-*1* mutant

As expected, the extraction of *B. distachyon* cell walls with NaOH after CDTA released primarily hemicelluloses, namely (1→3;1→4)-β-d-glucans (mixed-linkage glucans, MLG), xylans and xyloglucans (Fig. 4a). The antibody recognizing unsubstituted (1→4)-β-xylan (LM10) showed stronger signal in the *Bdlsi1*-*1* spikelets at the ripening stage as compared to the wild type, and a similar trend was observed in the entire mature shoots of *Bdlsi1*-*1* plants (Fig. 4a). At the ripening stage, the signal for the MLG epitope was higher in the stems and lower in the spikelets of the *Bdlsi1*-*1* mutant compared to the wild type, whereas at maturity, the abundance was relatively lower in both the straw and entire shoots of *Bdlsi1*-*1* (Fig. 4a). Using a specific assay to quantify MLG, we observed that at the ripening stage MLG was present in much higher concentrations in the spikelets than in the leaves and stems (Fig. 4b) In the latter two organs, the MLG concentration was similar and amounted to only approx. 9–14% of that in the spikelets (Fig. 4b). The MLG concentrations measured in stems and spikelets of the *Bdlsi1*-*1* mutant at the ripening stage were 30 and 34% lower than those in the wild type (Fig. 4b). From ripening to maturity, the MLG concentrations in all the samples increased up to several-fold (Fig. 4b, c). Moreover, at maturity, the MLG concentration was 42% higher in the *Bdlsi1*-*1* straw than in the wild type, while in the entire shoots, the wild type contained 70% more MLG than the *Bdlsi1*-*1* plants (Fig. 4c), the latter reflecting the higher MLG concentration in the spikelets of the wild type.

In grasses, arabinose residues of arabinoxylans (AX) can be modified via binding of ferulic acid (FA) that may bridge two adjacent AX molecules, but also cross-link hemicelluloses and lignin [48]. The presence of FA in the samples was detected by the LM12 antibody recognizing epitopes of polymers modified with FA. The signal for the LM12 antibody was detected only in the CDTA fraction, and the relative intensity observed in the mutant leaves and stems sampled at the ripening stage was lower compared to the wild type (Fig. 3a). The analysis of the mature material showed no signal for the LM12 antibody in the straw samples (Fig. 3a). However, an LM12 signal was obtained in the entire shoots, where the wild type showed much higher relative abundance than *Bdlsi1*-*1* (Fig. 3a). To further explore this difference, we performed immunolocalization using the LM12 antibody. While we did not succeed to obtain images for leaves, immunolabeling of the stem sections in the wild type revealed a strong fluorescence signal in the lignified sclerenchyma cells and in the xylem vessels (Fig. 4d). The corresponding tissues in the *Bdlsi1*-*1* mutant showed an overall reduction of the LM12 signal although with a relative intensification of the signal appearing in less lignified parenchymatic cells (Fig. 4d).

### Cellulose level in the straw is slightly elevated in the *Bdlsi1*-*1* mutant compared to the wild type

**Fig. 5:**
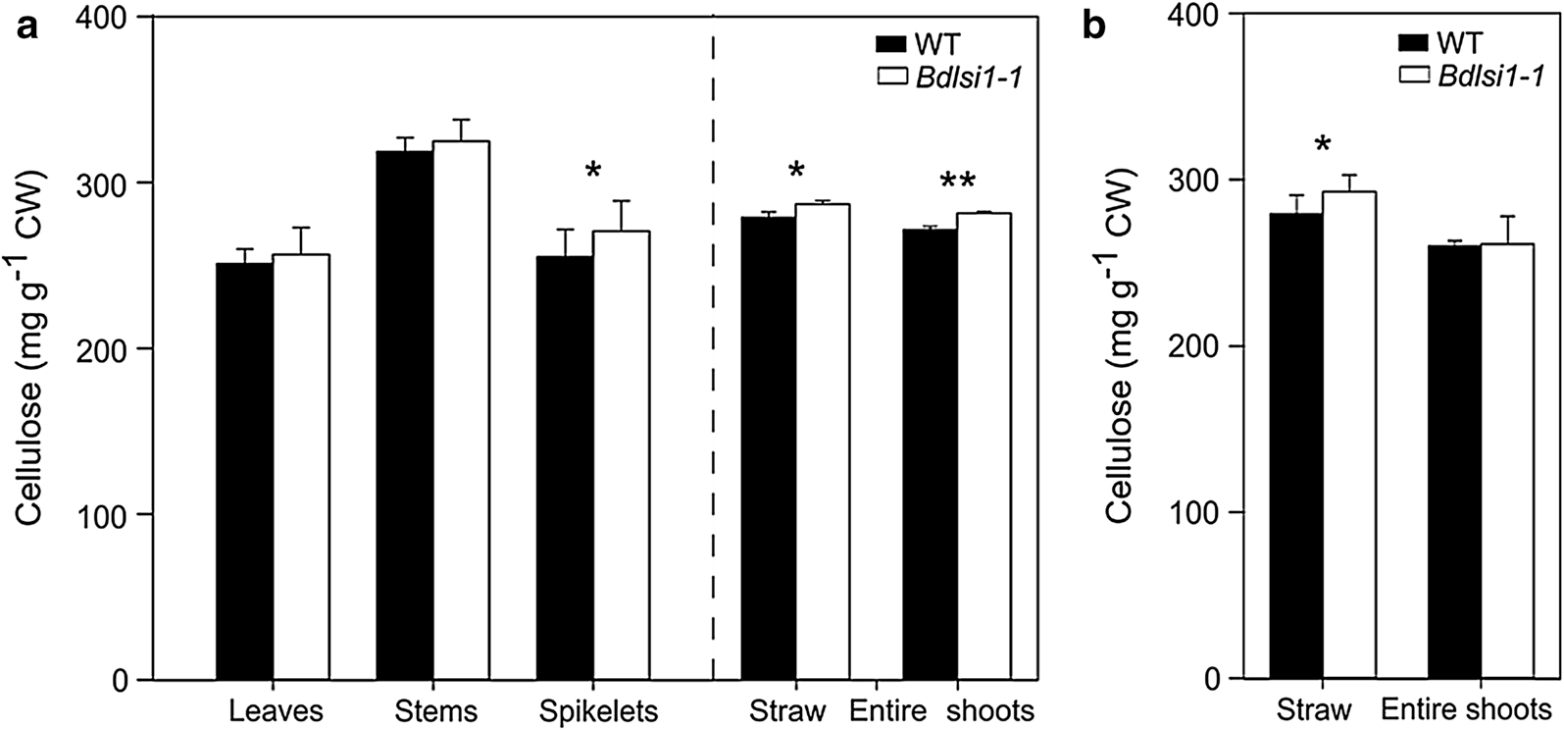
Concentrations of cellulose across organs and developmental stages in the wild-type and *Bdlsi1*-*1* mutant. The concentration of intact cellulose microfibrils in the TFA-resistant pellet was quantified in the wild-type (WT) and the *Bdlsi1*-*1* plants sampled at the ripening stage (**a**) and maturity (senescence) (**b**). Mean values (± SD) of three replicates are presented, asterisks indicate significant differences between the wild type and the mutant (*p ≤ 0.05; **p ≤ 0.01) as assessed by one-way ANOVA followed by Tukey’s HDS post hoc test

The residual pellet after TFA hydrolysis of cell wall material consists mostly of intact cellulose fibrils. At the ripening stage, the wild type and the mutant plants had comparable concentrations of TFA-resistant cellulose in leaves and stems, while the concentration measured in the *Bdlsi1*-*1* spikelets was 6% higher than that in the wild type (Fig. 5a). The cellulose concentration in the straw at both the ripening growth stage and at maturity was slightly, but significantly higher in the *Bdlsi1*-*1* mutant compared to the wild type (Fig. 5a, b). The same was the case for entire shoots at the ripening stage (Fig. 5a), but not at maturity (Fig. 5b).

### The *Bdlsi1*-*1* mutant exhibits minor changes in lignin composition

**Fig. 6:**
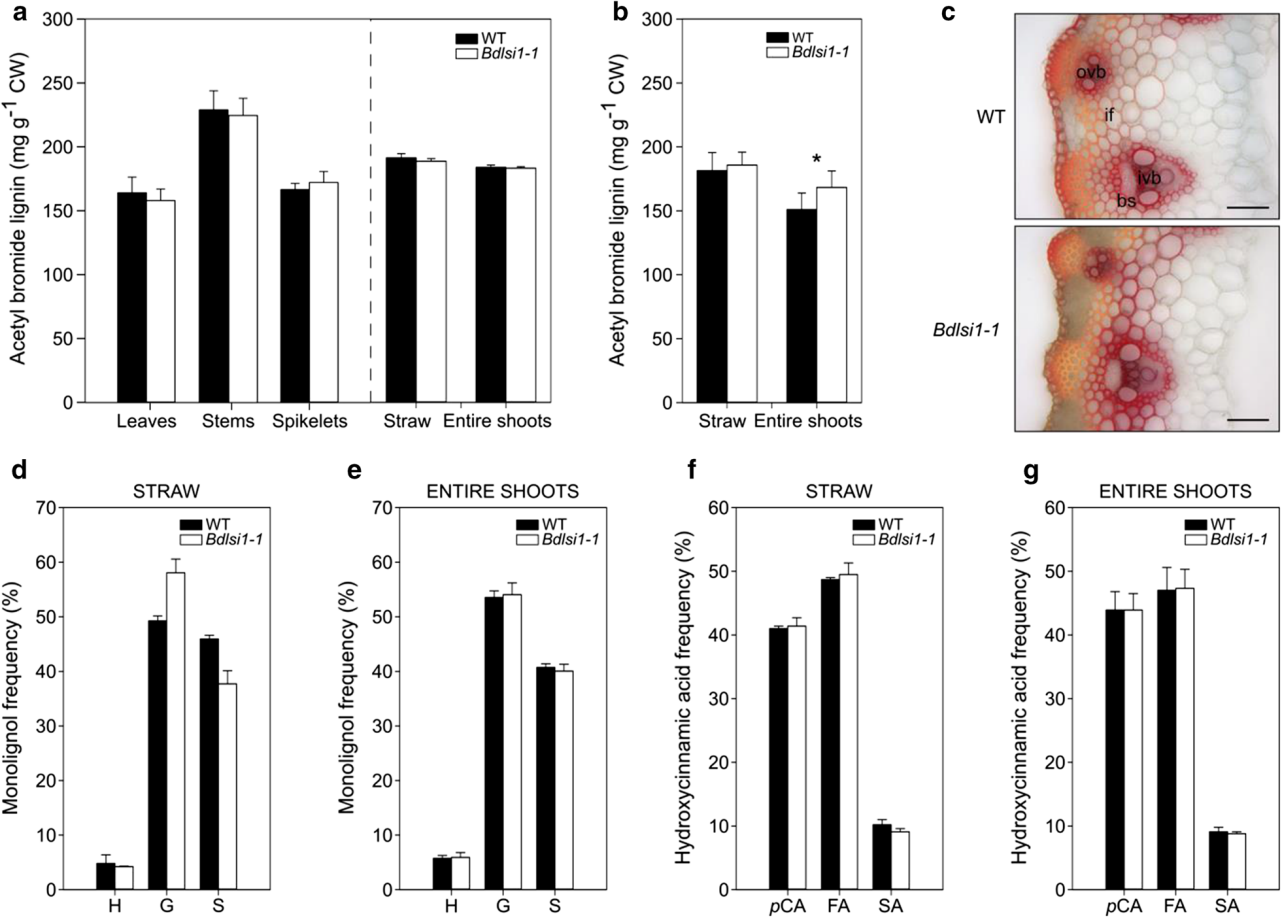
Characterization of lignin and hydroxycinnamates in wild type and *Bdlsi1*-*1* mutant plants. The concentrations of acetyl bromide soluble lignin were quantified in the wild-type and the *Bdlsi1*-*1* sampled at the ripening growth stage (**a**) and maturity (**b**). Results are presented as mean values (± SD, *n*  ≥  3), asterisk indicates statistically significant difference between the wild-type and the mutant plants (*p* ≤ 0.05), as assessed by one-way ANOVA followed by Tukey’s HDS post hoc test. **c** Lignin distribution in transverse sections from wild-type and *Bdlsi1*-*1* internodes sampled at the ripening stage was visualized with phloroglucinol staining (Wiesner method) (colour from light orange to red). Scale bars: 50 μm. Composition of monolignols was characterized in mature straw (**d**) and entire shoots (**e**) using analytical pyrolysis GC/MS. Data are presented as monolignol frequency (%), which is interpreted as an abundance of given monolignols in relation to all released monolignols. The composition of hydroxycinnamic acids in mature straw (**f**) and entire shoots (**g**) was quantified analogously to the lignin composition. Mean values (± SD, *n* ≥ 2) are presented. *ovb* outer vascular bundles, *ivb* inner vascular bundles, *if* interfascicular region, *bs* bundle sheaths, *S* syringyl unit, *G* guaiacyl unit, *p*-hydroxyphenyl unit, *FA* ferulic acid, *p-CA p*-coumaric acid, *SA* sinapic acid

To study lignin accumulation in the tissues of *B. distachyon,* we first quantified acetyl bromide soluble lignin. At the ripening stage, the lignin concentrations varied between the organs (Fig. 6a). The highest concentration was measured in the stems, where lignin constituted around 22% of the cell walls (Fig. 6a). The lignin concentrations in all the organs sampled at the ripening stage of growth were comparable between the wild type and the mutant (Fig. 6a). At maturity, the lignin concentration in the straw was similar for the wild type and mutant and similar to the values in the straw at the ripening growth stage (Fig. 6a, b). On the other hand, the entire shoots of *Bdlsi1*-*1* plants sampled at maturity contained significantly more lignin than the wild-type plants (Fig. 6b). Thus, the mature plants of the mutant contained relatively more lignin in the spikelets compared with the wild type.

Lignin quantification using the acetyl bromide method showed that stems were highly lignified organs in *B. distachyon*. To compare the distribution of lignin in stems of the *Bdlsi1*-*1* and the wild-type plants, we used histochemical staining with phloroglucinol (Wiesner method) (Fig. 6c). The phloroglucinol stain reacts with the *p*-hydroxycinnamaldehyde end-groups occurring in native lignin (mainly as coniferyl aldehyde end-groups) to give a reddish-purple colour, resulting in pale yellow staining of regions with low lignification (low quantity of *p*-hydroxycinnamaldehyde end-groups) and increasing orange/red staining as lignification proceeds (high proportion of *p*-hydroxycinnamaldehyde end-groups) [49]. We detected the highest level of lignification in the xylem cell walls, bundle sheaths and interfascicular fibres (Fig. 6c). Stems of the *Bdlsi1*-*1* mutant, collected at the ripening stage displayed increased red-hued staining relative to the wild type (Fig. 6c), thus indicating higher lignin level and/or higher abundance of coniferyl aldehyde end-groups in the lignin. This colour change appeared particularly in the interfascicular region (Fig. 6c).

To measure the lignin composition, we applied analytical pyrolysis (Py-GC/MS) that enables evaluation of pyrolytically labile monolignols and hydroxycinnamic acids. Only the samples harvested at maturity were analysed as plants here have undergone full lignification. G units were the most abundant in all the samples, followed by S units, accounting together for over 90% of all released monolignols (Fig. 6d, e). Small amounts of H units were also detected (Fig. 6d, e). Only the straw fraction differed in monolignol composition between the mutant and the wild type (Fig. 6d, e). The wild-type plants had comparable amounts of G and S units in the straw, whereas the *Bdlsi1*-*1* mutant had slightly higher content of S units and lower amount of G units than the wild type (Fig. 6d). These differences were not apparent in the entire shoots due to the influence of spikelets (Fig. 6d, e). All samples had similar profiles of released hydroxycinnamates with ferulic acid (FA) and *p*-coumaric acid (*p*-CA) monomers accounting for almost 90% of total hydroxycinnamates released (Fig. 6f, g).

### The saccharification efficiency of *Bdlsi1*-*1* mutant straw is roughly similar to that of the wild type

**Fig. 7:**
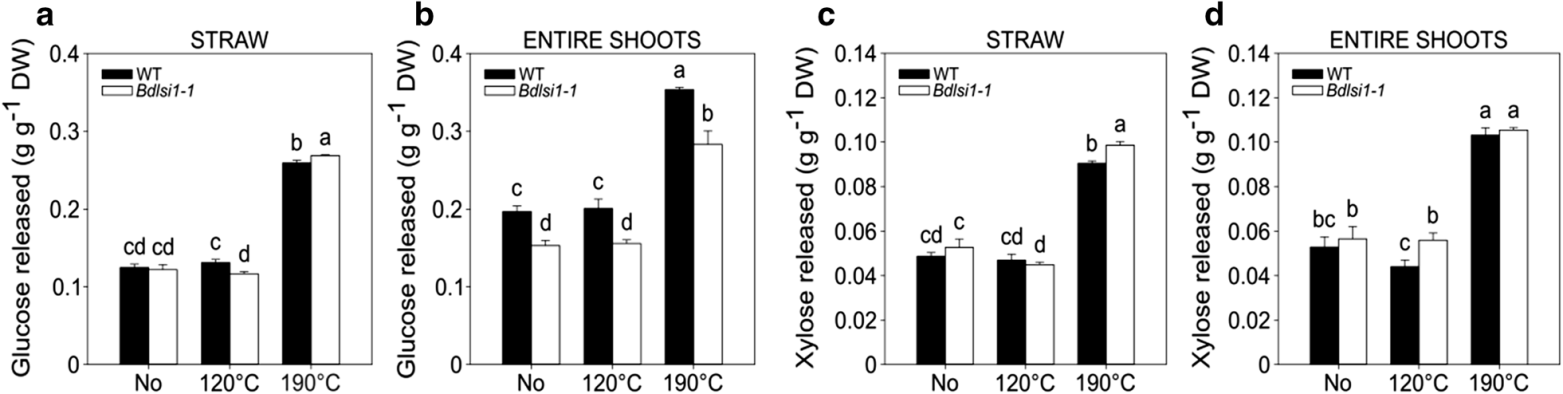
Glucose and xylose release from mature straw and entire shoots after enzymatic saccharification. The experiments were based on mature straw (**a**, **c**) or entire shoots (**b**, **d**) of the wild type (WT) and the *Bdlsi1*-*1* mutant. Three different pretreatment conditions were used, viz. no pretreatment, low severity (120 °C) and medium severity (190 °C) pretreatment. The saccharification efficiency was determined by release of glucose (**a**, **b**) and xylose (**c**, **d**) from pretreated and hydrolyzed material. Data presented are mean (± SD, *n* = 3); different letters indicate significant differences between the samples (*p* ≤ 0.05) as assessed by one-way ANOVA followed by Tukey’s HDS post hoc test

We tested the sugar release following enzymatic hydrolysis of straw and entire shoots of the wild-type and the *Bdlsi1*-*1* plants sampled at maturity (Fig. 7). Three biomass pretreatment conditions were included: (i) no pretreatment, (ii) low severity (120 °C) and (iii) medium severity (190 °C) pretreatment, the latter being typical for industrial hydrothermal pretreatment of straw. As expected, the low severity pretreatment (120 °C) did not improve sugar yield, whereas the more severe pretreatment (190 °C) resulted in approximately twofold higher sugar yields, compared to no pretreatment (Fig. 7). The entire shoots showed a higher release of glucose, but not xylose, as compared to the straw, most likely due to the contribution from starch and MLG in seeds that are more easily hydrolyzed than cellulose (Fig. 7). The increase in glucose yield between the straw and the entire shoots was more pronounced in the wild type, particularly when no pretreatment or mild pretreatment was applied (Fig. 7a, b). Pretreatment with 190 °C slightly, but significantly, improved both the glucose and xylose release from the *Bdlsi1*-*1* straw compared to that of the wild type (Fig. 7a, c). Finally, when the entire shoots were hydrolyzed, the release of glucose from the *Bdlsi1*-*1* plants was significantly reduced at all pretreatment conditions, which corresponds with the decreased concentration of glucose in the non-cellulosic fraction originating from breakdown of MLG (Figs. 2e, 4c and 7b).

## Discussion

### Low-Si status favours Si deposition in cell wall rather than in silicified structures

Comprehensive studies of *B. distachyon* demonstrate its utility in different areas of grass research, among other cell walls and saccharification potential [25, 50, 51]. Cell wall composition and the interactions of Si with cell wall components may play an important role for the value of lignocellulosic biomass for biofuel and biorefinery purposes. The occurrence of cell wall-bound Si is well documented in the literature, although the mechanism of deposition remains elusive [8, 9]. A substantial proportion of Si absorbed by plants ends up within the cell walls. This is, e.g. shown by the fact that cell walls isolated from rice cell suspension culture contained approximately 3 times higher Si concentration than in the whole cells [9]. In the shoots of wild-type *B. distachyon* harvested at the ripening stage, the majority of Si was present as phytoliths and only 9% of the total Si was non-phytolith Si present as amorphous Si and cell wall-bound-Si (Table 1 and Additional file 2: Figure S1). In contrast, the *Bdlsi1*-*1* mutant plants contained half of the total Si in non-phytolith form and the overall amount of phytoliths was substantially reduced in all organs (Table 1 and Additional file 2: Figure S1). This suggests that during limited Si availability, Si present in the amorphous form or directly bound with cell walls polysaccharides has priority over the formation of silicified structures. However, it remains unknown if this is mainly a result of a passive co-aggregation or also involves active regulation of transport and translocation pathways [52]. Cell wall B status was also affected by mutation in the *Bdlsi1*-*1* plants; the decrease, however, was less severe than for Si, and the difference disappeared at maturity (Fig. 1c, d). Both elements play an important role during growth and development and can affect the seed quality [29, 53, 54]. Consequently, low B and Si status in plant tissues may affect their allocation to favour deposition in the spikelets upon seed filling, as observed in our study (Table 1 and Additional file 3: Table S2).

### Silicon and cell wall components in straw

Silicon has been suggested to play a structural role in cell walls. In rice cell suspension cultures, Si was detected within cell walls, where it improved the mechanical properties and regeneration of cell walls in protoplasts [9]. Despite numerous evidences for Si involvement in the cell wall structure, most studies have focused on the functional roles of Si in plants exposed to stresses, while only few have looked into the role of Si under optimal growth conditions. Yamamoto et al. [55] reported enhanced secondary cell wall synthesis resulting in increased accumulation of cellulose and lignin in rice grown without Si supply. They suggested that the alterations compensated for reduced Si-based rigidity. Conversely, Zhang et al. [11] showed lower content of cellulose, hemicelluloses and lignin in rice grown without Si supply. Elevated cellulose concentrations, but not lignin, were observed in the straw of the *Bdlsi1*-*1* mutant grown in soil, but the increase (3–5%; Figs. 5, 6a, b) was not as prominent as in rice (50% in leaves). Since Si constituted < 0.1% of the mass of the cell walls (Fig. 1a, b), the increase in cellulose in the cell walls of the mutant was much greater than what could be explained by the relative decrease in Si.

In grasses, pectins account for a minor fraction of cell walls, but they greatly affect the integrity and properties of the cell wall network [20, 56]. Our observations suggest distinct extractability and organ-specific characteristics of HG and RG-I in the *B. distachyon* plants (Figs. 3a and 4a). Three different observations, viz. (i) the reduced concentration of GalA (Fig. 2a), (ii) the overall higher total degree of methylesterification (Fig. 3b), and (iii) the lower relative abundance of HG epitopes in the *Bdlsi1*-*1* leaves (Fig. 3a), suggest that there was a reduction in the level of HG in the mutant leaves and that some HG regions were more methylesterified. These changes (high methylesterification) would favour the hydration and flexibility of cell walls. On the other hand, the low extractability of HG and the decline in methylesterification in *Bdlsi1*-*1* stems (Fig. 3a, b) might indicate local cell wall stiffening via the formation of Ca-bridges between de-esterified HG residues, as also observed in response to reduced cellulose concentration in tobacco and *Arabidopsis thaliana* [57, 58]. The distinct changes observed in the pectin structure of the mutant might be a response to the lower Si concentration with different consequences for leaves and stems as part of an organ-specific remodelling mechanism compensating for the Si decline.

Mixed-linkage glucans have been proposed to interact with Si in plant cell walls, and possibly act as template for Si deposition [10, 59]. In rice plants, MLG participated in Si-based cell wall strengthening. Transgenic rice lines with reduced MLG content did not show differences in the total Si concentration but exhibited reduced mechanical strength and altered Si deposition pattern [10]. In grasses, high levels of MLG have been detected in primary cell walls at the very early stages of development and in the storage endosperm, but also in mature tissues (e.g. rice stems) [27, 60, 61]. In vegetative tissues, MLG were suggested to affect the cell wall composition of the expanding cells via influencing the organization of cellulose microfibrils and interactions with AX [62]. In the present study, the *B. distachyon* plants generally deposited lower amounts of MLG in the vegetative tissues compared to the seeds, but continued to accumulate MLG in all the organs between the ripening growth stage and maturity (Fig. 4b, c). At the ripening stage, the mutant had a relatively low amount of MLG in the straw but as the plants matured, the concentration exceeded that measured in the wild type. These alterations may affect cell wall properties and arrangement of polymers in the cell wall matrix.

The pattern and degree of substitutions of the xylan backbone may have a great impact on the structure of cell walls as they determine interactions with cellulose and other xylans [63]. Our observations suggest increased accumulation of AX in the mature mutant plants, but also an increase in the degree of substitution with Ara*f* (Figs. 2 and 3a). In addition, the lower extractability of FA from the *Bdlsi1*-*1* mutant (Fig. 3a) and the weaker immunolabeling of the mutant stem sections (Fig. 4d) indicate alterations in polymer cross-linking. Dimers of FA intra- and interlink molecules of AX and FA also form cross-links between AX and lignin [48].

The lignin units are connected through a range of C–O–C or C–C bonds; the composition and type of the linkages may vary greatly and affect the structure and properties of lignin [64]. In our study, however, the lignin fraction of the *Bdlsi1*-*1* mutant showed only minor changes compared to the wild type (Fig. 6). It appeared that the lignin content was not altered, but minor changes in the composition of pyrolytically labile monolignols as well as differences in lignin staining, nevertheless, indicate alterations of the polymer structure.

Taken together our observations suggest that a low concentration of Si in the straw of *B. distachyon* mostly triggers compositional changes of the cell wall network, such as types of linkages and structural rearrangements of polymers, rather than affecting cell wall thickness.

### Silicon and cell wall components in seeds

Silicon fertilization is commonly used in rice production as it positively affects seed quality and yield [65]. We have also shown that the availability of exogenously supplied Si affected the formation of seeds in *B. distachyon* and that Si defiency caused lower seed weights [29]. Compositional analysis of the mutant cell walls revealed a significant decrease in the MLG concentration in the cell walls of the spikelets (Fig. 4b). The cell walls of the cereal endosperm are characterized by a high abundance of MLG and AX substituted with FA, but the relative proportion of AX to MLG varies between plant species [66]. *B. distachyon* is a wild grass; therefore, it accumulates high amounts of cell wall polysaccharides, mainly MLG, in the endosperm and, as opposed to domesticated cereals, only small quantities of starch [60]. In that context, MLG were proposed to function as storage polysaccharides to be remobilized during seed germination. Arabinoxylans were also detected in the seeds of *B. distachyon*, accounting for approximately 4.7% of the weight of de-hulled seeds [60]. The high arabinose and xylose concentration in the spikelets observed in the present work (Fig. 2c) thus likely represent a contribution from the bracts.

Relative to the wild type, the mutant spikelets showed a low Ara/Xyl ratio and an increase in the signal intensity of the antibody LM10 recognizing unsubstituted xylans (Figs. 2c and 4a). These alterations may decrease the solubility of AX and favour the formation of hydrogen bonds between xylan chains. At the same time, the concentration of cellulose in the *Bdlsi1*-*1* spikelets at the ripening stage was elevated (Fig. 5a). The *B. distachyon* seed contains about 6% cellulose in the cell walls distributed across the whole seed although most abundantly in the cell walls of the outer seed layers [60]. In our study, cellulose accounted for approx. 25% of the spikelet cell walls, thus, indicating that the majority derived from the bracts surrounding the seed (Fig. 5a). Increased cellulose in the *Bdlsi1*-*1* spikelets might be a result of seed cell wall thickening and/or fortification of the bracts in the absence of Si.

### Silicon, cell wall composition and saccharification efficiency

Cell wall polysaccharides are substrates for enzymatic conversion into a range of monomers and oligomers. The heterogeneity of the macromolecules and lignin comprising the cell walls, their complex organization into a 3D network and the various types of interactions among individual building blocks impose recalcitrance to enzymatic hydrolysis [64, 67]. The presence of Si is another crucial factor to take into account when considering the use of lignocellulosic biomass for bioenergy and biorefining purposes. Silicon may not only have a negative effect on the durability of equipment used during conversion, but may also decrease the energy output during conversion as well as increase the recalcitrance of the biomass [18, 68]. Despite the contrasting Si concentrations and the marked differences in the cell wall network between the wild-type and the *Bdlsi1*-*1* plants, there was only a small improvement of the glucose and xylose yield following standard pretreatment and saccharification of the mature straw (Fig. 7a, c). Thus, a reduction of the Si supply and the Si concentration within the biomass of *B. distachyon* seems to have a limited effect on the cell wall recalcitrance. This suggests that loss of Si-mediated cell wall strengthening is compensated for by rearrangements in the cell walls maintaining the inherent recalcitrance of biomass.

## Conclusion

In this study, we have characterized the deposition of Si and the cell wall composition in wild-type and *low*-*silicon 1* (*Bdlsi1*-*1*) mutant plants of *Brachypodium distachyon*. We conclude that low Si availability leads to remodelling of the structure and linkages of cell wall components. The most affected cell wall components are pectins, hemicelluloses and lignin. However, the rearrangements of the cell wall network seem not to significantly alter the innate recalcitrance of cell walls towards enzymatic saccharification.

## Additional files

**Additional file 1: Table S1.** Monoclonal antibodies and carbohydrate binding modules used in the study.

**Additional file 2: Figure S1.** Phytoliths in *Brachypodium distachyon* wild type and *Bdlsi1*-*1* mutant. Phytoliths remaining after microwave-assisted acid digestion of plant material (AIR) were recovered from filters, labelled with silica-specific PDMPO dye and observed by light and fluorescence microscopy. Leaves (A, B, C), stems (D, E) and spikelets (F, G) of wild type plants showed high density of silicified structures that varied in the intensity of PDMPO-labelling. In contrast, the leaves (H, I, J), stems (K, L, M) and spikelets (N, O, P) of the mutant plants contained a substantially lower amount of phytoliths. Scale bars: 100 µm.

**Additional file 3: Table S2.** Boron distribution among organs and their cell walls in wild type and mutant plant harvested at the ripening stage. Data show the average B content in organs of dry wild type and mutant plants and the proportion of this B present in cell walls (CW). Boron concentrations were measured by inductively coupled plasma optical emission spectrometry (ICP-OES) (*n* = 3). The standard deviation of means used in the calculations did not exceed 20% of the mean value.

## Abbreviations

AIR: alcohol insoluble residue
AX: arabinoxylan
AGP: arabinogalactan protein
B: boron
Ca: calcium
CDTA: 1,2-diaminocyclohexanetetraacetic acid
CoMPP: comprehensive microarray polymer profiling
CW: cell wall
DM: methylesterification degree
DW: dry weight
FA: ferulic acid
GalA: galacturonic acid
GlcA: glucuronic acid
HC: hemicelluloses
HG: homogalacturonan
ICP-OES: inductively coupled plasma optical emission spectrometry
LSI: silicon transport proteins
MLG: mixed-linkage glucans
*p*CA: *p*-coumaric acid
PBS: phosphate buffered saline
PDMPO: 2-(4-piridyl)-5-((4-(2-dimethylaminoethylaminocarbamoyl)-methoxyl)phenyl)oxazole
RG-I: rhamnogalacturonan-I
Si: silicon
TFA: trifluoroacetic acid
