# Supplementary material for: The impact of silicon on cell wall composition and enzymatic saccharification of Brachypodium distachyon
Source: Biotechnol Biofuels. 2018 Jun 20;11:171. doi: 10.1186/s13068-018-1166-0 (PMC6009033; doi:10.1186/s13068-018-1166-0)
Supplement: Supplementary file 1 — Additional file 1: Table S1. Monoclonal antibodies and carbohydrate binding modules used in the study. [file 13068_2018_1166_MOESM1_ESM.pdf]

| **Epitope** | **Antibody** | **References** |
| --- | --- | --- |
| HG partially/de-esterified | JIM5 | (Verhertbruggen et al., 2009) |
| HG partially esterified | JIM7 | (Verhertbruggen et al., 2009) |
| HG partially/de-esterified | LM18 | (Verhertbruggen et al., 2009) |
| HG partially/ de-esterified | LM19 | (Verhertbruggen et al., 2009) |
| HG partially esterified | LM20 | (Verhertbruggen et al., 2009) |
| Xylogalacturonan | LM8 | (Willats et al., 2004) |
| RG-I backbone | INRA-RU1 | (Ralet et al., 2010) |
| (1→5)-α-arabinan | LM6 | (Willats et al., 1998) |
| Feruloylate on polymers | LM12 | (Pedersen et al., 2012) |
| (1→4)-β-D-(galacto)(gluco)mannan | LM21 | (Marcus et al., 2008) |
| (1→3; 1→4)-β-glucan (MLG) | BS-400-3 | (Meikle et al., 1994) |
| Xyloglucan | LM24 | (Pedersen et al., 2012) |
| Terminal (1→4)-β-D-xylan | LM23 | (Pedersen et al., 2012) |
| Extensin | LM1 | (Smallwood et al., 1995) |
| Extensin | JIM11 | (Smallwood et al., 1994) |
| Extensin | JIM12 | (Smallwood et al., 1994) |
| Extensin | JIM19 | (Smallwood et al., 1994) |
| AGP | JIM4 | (Yates and Knox, 1994; Yates et al., 1996) |
| AGP | JIM15 | (Yates and Knox, 1994; Yates et al., 1996) |
| AGP | JIM16 | (Yates and Knox, 1994; Yates et al., 1996) |
| AGP | JIM17 | (Pattathil et al., 2010) |
| AGP | LM14 | (Moller et al., 2008) |
| (1→4)-β-D-xylan/arabinoxylan | LM11 | (McCartney et al., 2005) |
| Extensin | JIM20 | (Smallwood et al., 1994) |
| AGP | JIM13 | (Yates et al., 1996) |
| AGP; β-linked GlcA | LM2 | (Yates et al., 1996) |
| (1→4)-β-D-galactan | LM5 | (Jones et al., 1997) |
| Linearised (1→5)-α-arabinan | LM13 | (Moller et al., 2008) |
| Xyloglucan (XXXG) | LM15 | (Marcus et al., 2008) |
| Xyloglucan | LM25 | (Pedersen et al., 2012) |
| (1→4)-β-D-xylan | LM10 | (McCartney et al., 2005) |
