# Supplementary figures and images for: The impact of silicon on cell wall composition and enzymatic saccharification of Brachypodium distachyon
Source: Biotechnol Biofuels. 2018 Jun 20;11:171. doi: 10.1186/s13068-018-1166-0 (PMC6009033; doi:10.1186/s13068-018-1166-0)

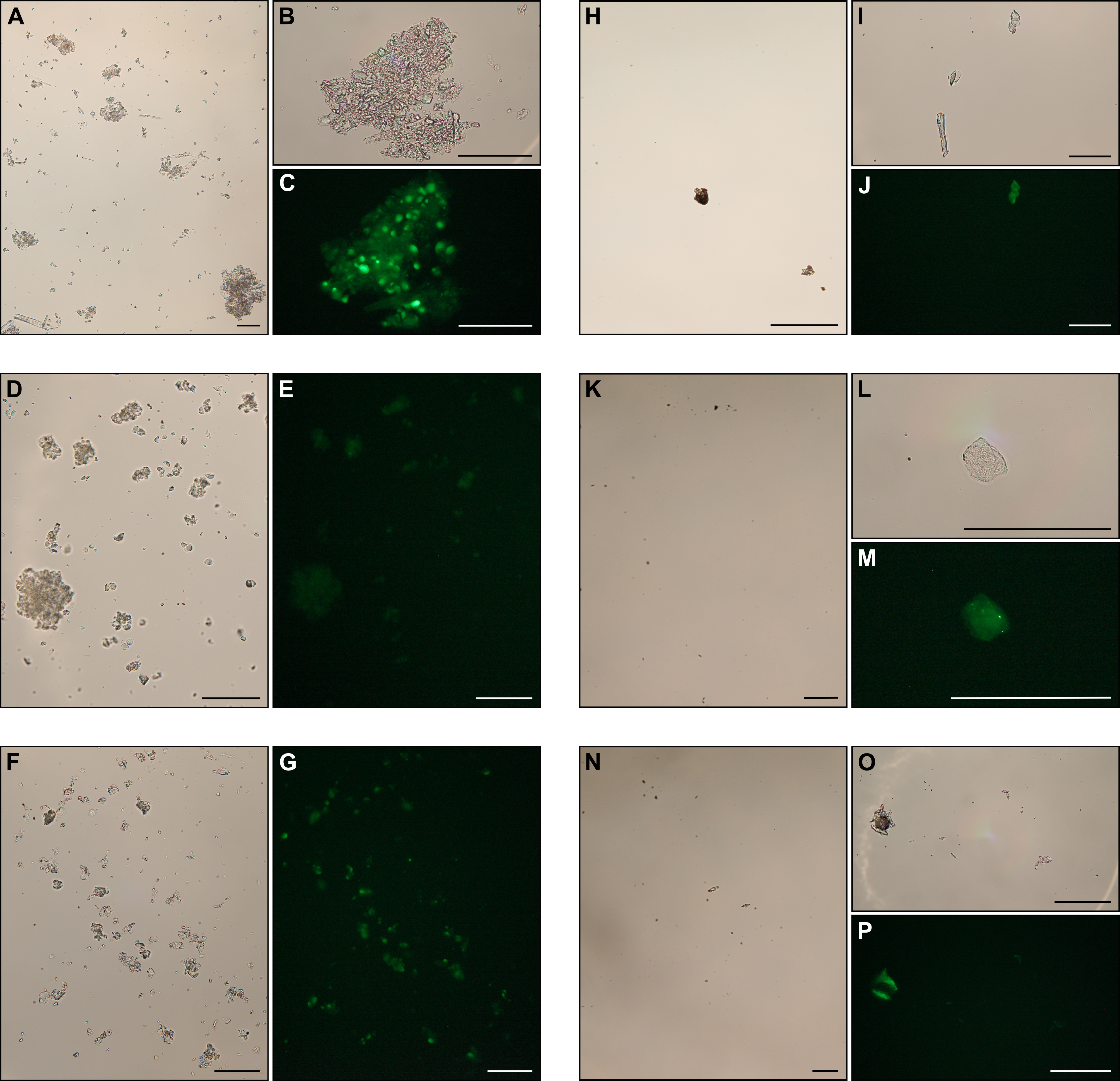

Supplement: Supplementary file 2 — Additional file 2: Figure S1. Phytoliths in Brachypodium distachyon wild type and Bdlsi1-1 mutant. Phytoliths remaining after microwave-assisted acid digestion of plant material (AIR) were recovered from filters, labelled with silica-specific PDMPO dye and observed by light and fluorescence microscopy. Leaves (A, B, C), stems (D, E) and spikelets (F, G) of wild type plants showed high density of silicified structures that varied in the intensity of PDMPO-labelling. In contrast, the leaves (H, I, J), stems (K, L, M) and spikelets (N, O, P) of the mutant plants contained a substantially lower amount of phytoliths. Scale bars: 100 µm. [file 13068_2018_1166_MOESM2_ESM.png]
