# Supplementary material for: The impact of silicon on cell wall composition and enzymatic saccharification of Brachypodium distachyon
Source: Biotechnol Biofuels. 2018 Jun 20;11:171. doi: 10.1186/s13068-018-1166-0 (PMC6009033; doi:10.1186/s13068-018-1166-0)
Supplement: Supplementary file 3 — Additional file 3: Table S2. Boron distribution among organs and their cell walls in wild type and mutant plant harvested at the ripening stage. Data show the average B content in organs of dry wild type and mutant plants and the proportion of this B present in cell walls (CW). Boron concentrations were measured by inductively coupled plasma optical emission spectrometry (ICP-OES) (n = 3). The standard deviation of means used in the calculations did not exceed 20% of the mean value. [file 13068_2018_1166_MOESM3_ESM.pdf]

Organ	Plant	Total B in dry matter (µg)	B (% of total B in DW)	Total B in cell walls (µg)	B (% of total B in CW)	Ratio of B in CW to B in DW (%)
**Leaves**	**WT**	64	70	19	64	29
	Bdlsi1-1	23	51	6	46	28
**Stems**	**WT**	3	3	2	7	73
	Bdlsi1-1	3	7	3	20	87
**Spikelets**	**WT**	24	26	8	29	34
	Bdlsi1-1	20	43	5	34	24
**Entire plant**	**WT**	91	100	29	100	32
	Bdlsi1-1	46	100	14	100	30
